# Superselective embolisation for control of intractable epistaxis from maxillary artery injury

**DOI:** 10.2349/biij.7.1.e3

**Published:** 2011-01-01

**Authors:** P Singam, J Thanabalan, Z Mohammed

**Affiliations:** 1 Department of Surgery, Universiti Kebangsaan Malaysia Medical Centre, Kuala Lumpur, Malaysia; 2 Department of Radiology, Universiti Kebangsaan Malaysia Medical Centre, Kuala Lumpur, Malaysia

**Keywords:** Epistaxis, maxillary artery injury, head trauma

## Abstract

Traumatic intractable epistaxis following fractures of the facial and base of skull rarely may be life-threatening. Common sites of injury are the internal carotid and maxillary artery. When conventional methods of arresting haemorrhage fail, the choices are then an open arterial ligation or superselective embolisation. This paper presents a patient with life-threatening epistaxis from a Le Fort type II fracture. Angiography revealed a maxillary artery injury in which superselective embolisation was performed and the haemorrhage was successfully arrested. A literature review of this technique is discussed, including its advantages and the relationship of the internal maxillary artery to facial fractures.

## INTRODUCTION

Life-threatening haemorrhage, including epistaxis, secondary to head trauma may occur in up to 11% of trauma cases [1]. The incidence of intractable epistaxis secondary to trauma occurs in 5% of all cases of severe epistaxis [2]. This could lead to compromised airways, haemorrhagic shock, disseminated intravascular coagulopathy, hypoxia and secondary brain injury. Intractable epistaxis usually results from an injured internal carotid or maxillary artery. In its treatment, when all conservative measures such as nasal packing, cauterisation and surgical vessel ligation fail, superselective angiography and subsequent embolisation is the treatment of choice.

## CASE REPORT

A 26-year-old man was involved in a motor vehicle accident and suffered severe facial injury. His Glasgow Coma Scale (GCS) was 6/15 and he had massive bleeding from his ears, nose and oral cavity. His blood pressure (BP) on admission was 104/40 mmHg, heart rate (HR) was 110 per min. Lung auscultation revealed generalised crepitations. There were no scalp lacerations, neck deformities or bruising. He had considerable facial swelling, and a detailed maxillo-facial examination was deferred. Within an hour his BP had dropped to 70/30 mmHg, HR was 118 per min, and oxygen saturation (SpO2) and GCS had deteriorated to 50% and 3/15, respectively. He was intubated, ventilated and fluid resuscitation with colloids and crystalloids was vigorously infused. In view of the persistent epistaxis, bilateral anterior and posterior nasal packing was done. Despite this, the epistaxis was persistent, resulting in an estimated 3 litres of blood loss four hours after trauma. He had been hypotensive throughout this period. Three pints of packed cell blood was transfused. A computer tomography (CT) scan of the brain revealed the presence of pneumocranium and swollen brain parenchyma with basal cisterns obliteration. There was a fracture on the base of skull involving the right sphenoid wing and maxillary sinuses without intracranial haemorrhage. With a diagnosis of diffuse axonal injury and massive epistaxis secondary to the base of skull fracture, he was admitted and treated with standard neuroprotective therapy in the Intensive Care Unit.

His haemoglobin level was 7.4 g/dl with deranged coagulation profile for which 6 units of fresh frozen plasma (FFP), 4 units of platelets, 6 units of cryopercipitates and a further 3 pints of blood was transfused. Bilateral anterior and posterior nasopharyngeal packing using the inflated Foley catheter balloon was performed but the epistaxis was still persistent. Suspicion of a major arterial injury from the base of skull fracture was considered and angiography was performed 18 hours post-trauma.

**Figure 1:**
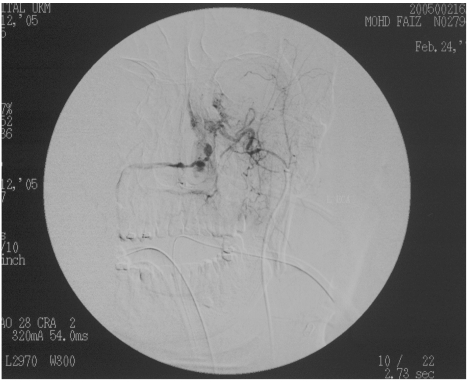
Anterior posterior view showing extravasation of contrast across the nasal cavity from the injured left maxillary artery

**Figure 2:**
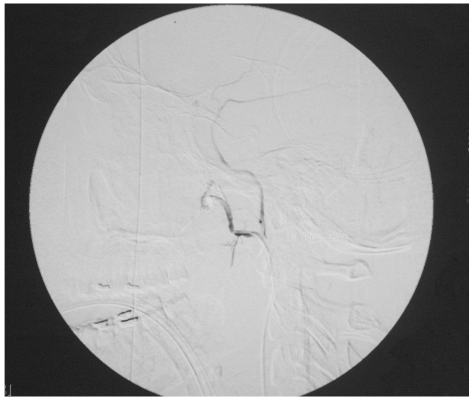
Lateral view post-embolisation with polyvinyl alcohol particles (PVA). Note absent contrast extravasations and complete arrest in haemorrhage

The right femoral artery was cannulated with an 18J 7 cm needle 5F sheath and a 5F head hunter catheter. Both the external carotid and right internal carotid arteries were cannulated, with the internal maxillary arteries also selectively cannulated. Angiography revealed active bleeding in the nasal cavity and nasopharynx, following selective left internal maxillary artery (IMA) runs (Figure 1). The branch from the left internal maxillary artery demonstrated pseudo aneurysm formation. The internal carotid arteries were normal. Both right and left IMAs were embolised. In view of the patient’s condition and failure of conservative measures, permanent embolic material, Polyvinyl Alcohol (PVA) particles were used for the left IMA. Despite not demonstrating active bleed from the right IMA, non-permanent particles (gelfoam) were used for embolisation of the right IMA to prevent any re-bleeding which could occur later. Post-embolisation runs showed arrest of contrast extravasation (Figure 2). Five hours post-embolisation, the epistaxis had been completely arrested and his blood pressure had normalised to 135/72 mmHg. At 24 hours post-trauma, he had received a total of 11 pints of blood, 6 units of FFP, 12 units of cryopercipitates and 6 units of platelets. Over the next 48 hours, he became normotensive with return of normal heart rate. The throat and nose packs together with Foley’s catheter were removed. A three-dimensional CT scan of the skull revealed a Le Fort II fracture involving the bilateral maxilla and nasal bridge, as well as a left zygomatic complex fracture, and fracture of the right condyle of the mandible and left angle of the mandible.

## DISCUSSION

Intractable epistaxis following trauma can occur immediately or as a delayed reaction. Common sources of bleeding include the IMA, spheno-palatine artery and internal carotid artery. The pathology may involve direct trauma and disruption of vessel or formation of a pseudoaneurysm.

The internal maxillary artery supplies the deep structure of the face and some of the meninges. It is a branch of the external carotid artery and enters the intratemporal fossa by passing forwards deep to the neck of the mandible, then passes between two heads of the lateral pterygoid muscle, into the pterygoimaxillary fissure and then into the pterygopalatine fossa. It is divided into three parts, before, on and beyond the lateral pterygoid muscle [3].

Our patient suffered a Le Fort II fracture involving the bilateral maxilla and nasal bridges, a left zygomatic complex fracture, as well as fractures of the right condyle of the mandible, left angle of the mandible, left sphenoid wing and maxillary sinus. The left maxillary artery, with its branches, are in close proximity to these structures and therefore very likely to be directly injured.

Conservative measures to control intractable epistaxis from a major arterial bleed, including nasal packing, anatomical reduction of mid-facial fracture or rigid fixation, are usually only transient while resuscitation is on-going. Further management entails invasive interventions like arterial ligation or selective embolisation. Arterial ligation to the external carotid or maxillary artery is technically demanding and difficult to perform in acute situations where general anaesthesia is required with a haemodynamically unstable patient [4, 6]. Furthermore, the effect of arterial ligation is variable because of collateral circulation from the contra-lateral side and communication with the internal carotid artery [4].

Angiography and selective embolisation to detect and control traumatic arterial injury is less invasive and faster than open arterial ligation [5]. Its advantage is that it is able to demonstrate the feeding vessels and localise the exact anatomic sites of bleeding, offers more distal access to bleeding points, avoids the need for general anaesthesia, requires a short time for the procedure and allows preservation of other branches of external carotid artery. Even if therapeutic embolisation is not feasible, angiogram delineates the vessel architecture thus allowing surgical intervention to be accurately planned [4, 6].

Embolisation of the internal maxillary artery for intractable epistaxis was first reported in 1974 by Sokoloff et al. Since then, there reports of this technique have been published, with success rates varying from 71% to 100% [5, 6]. In the majority of reported cases, superselective embolisation was used for ruptured pseudoaneurysms of IMA. In these cases, epistaxis occurred at variable time periods and were either post-trauma, after radiation or resulting from tumours. Ultimately, the resolution of haemorrhage is often immediate.

Complications related to this method include cerebrovascular accidents (CVA), hemiplegia, blindness, facial nerve palsy, seizures and soft tissue necrosis. The overall complication rate in published reports is around 3–27%. The majority of complications reported were minor and transient [5].

## CONCLUSION

With the ability to acquire multi-modality imaging in an emergency setting, future cases of intractable epistaxis resulting from trauma should prompt earlier decisions to undergo interventional imaging, as the source is almost always a major arterial bleed. The high success rates and low complication rates would justify its use early in future protocols for the management of post-traumatic severe epistaxis, and thus minimise the associated morbidity and mortality.
